# Long-term safety and efficacy of endovascular ultrasound renal denervation in resistant hypertension: 8-year results from the ACHIEVE study

**DOI:** 10.1007/s00392-024-02555-7

**Published:** 2024-10-23

**Authors:** Victor J. M. Zeijen, Sebastian Völz, Thomas Zeller, Felix Mahfoud, Michael Kunz, Karl-Heinz Kuck, Bert Andersson, Tobias Graf, Horst Sievert, Philipp Kahlert, Meital Horesh-Bar, Mattie J. Lenzen, Isabella Kardys, Joost Daemen

**Affiliations:** 1 Department of Cardiology, Thoraxcenter, Erasmus University Medical Center, Room Rg-628, P.O. Box 2040, 3000 CA Rotterdam, The Netherlands; 2 Department of Cardiology, Sahlgrenska University Hospital, Gothenburg, Sweden; 3 Department of Angiology, University Heart Center Freiburg-Bad Krozingen, Bad Krozingen, Germany; 4 Clinic of Cardiology, Angiology and Intensive Care Medicine, Internal Medicine III, Saarland University Hospital, Saarland University, Homburg, Germany; 5 Department of Cardiology, University Heart Center Lübeck, University Hospital Schleswig-Holstein, Lübeck, Germany; 6 Cardiovascular Center Frankfurt, Frankfurt, Germany; 7 Department of Cardiology and Vascular Medicine, University Clinic Essen, West-German Heart and Vascular Center, Essen, Germany; 8 ReCor Medical Inc, Palo Alto, CA USA

**Keywords:** Antihypertensive agents, Blood pressure monitoring, Ambulatory, Glomerular Filtration Rate, Hypertension, Renal artery, Sympathectomy

## Abstract

**Background:**

Ultrasound renal sympathetic denervation (uRDN) reduces blood pressure (BP) in the absence and presence of antihypertensive treatment at 2 months. Beyond 3 years, there is a lack of follow-up data. This study investigated the long-term safety and efficacy of uRDN.

**Methods:**

This prospective observational study recruited patients previously included in the international multicenter ACHIEVE study, with office systolic blood pressure (SBP) ≥160 mmHg, 24 h ambulatory SBP ≥130 mmHg, ≥3 antihypertensive drugs and *estimated Glomerular Filtration Rate* (eGFR) ≥45 ml/min/1.73m^2^ undergoing uRDN. The primary efficacy outcome was 24 h ambulatory SBP, adjusted for the number of defined daily dosages (DDD) of antihypertensive drugs. Statistical analyses were performed using linear mixed-effects models and inverse probability weighting.

**Results:**

A total of 27 out of the initially enrolled 96 patients underwent prospective follow-up at a median of 8.2 [7.6−8.9] years. Mean age was 62.6±9.3 years (37.0% female). Preprocedural 24 h ambulatory BP was 151.9/84.1±11.5/11.1 mmHg and the median number of DDDs was 5.0 [4.3−7.0]. At 8 years after uRDN, the change in 24 h ambulatory SBP was −19.5 [95%CI −26.7,−12.4] mmHg (*p*<0.001). The 8-year change in the number of DDDs was −1.7 [−2.8,−0.6] (*p* = 0.003). The 8-year decline in eGFR was −8.9 [−13.2,−4.7] ml/min/1.73m^2^ (*p*<0.001). Clinical event data were available for all 96 patients (median follow-up 3.5 [1.0–8.0] years). Renal failure occurred in one patient and no cases of renal artery stenosis were detected.

**Conclusions:**

A significant BP reduction was observed up until 8 years following uRDN in parallel to a decrease in drug burden over time, in the absence of procedure-related adverse events.

**Supplementary Information:**

The online version contains supplementary material available at 10.1007/s00392-024-02555-7.

## Introduction

Antihypertensive treatment significantly reduces cardiovascular risk by 10% to 20% per 5 to 10 mmHg reduction in systolic blood pressure (SBP), respectively [1, 2]. Unfortunately, these figures have been challenged by the inability to maintain durable blood pressure (BP) control in up to 50% of patients, which has been directly linked to an increase in cardiovascular risk, despite the availability of a broad armamentarium of antihypertensive treatment modalities [3–5].

Throughout the last decade, renal sympathetic denervation (RDN) has emerged as an invasive treatment modality for patients with uncontrolled hypertension. Within a 2-month time horizon, ultrasound renal denervation (uRDN) demonstrated to significantly lower ambulatory SBP by 4 to 6 mmHg as compared to sham-control, both in patients with and without antihypertensive drug therapy [6–9].

The trAnsCatHeter Intravascular ultrasound Energy deliVery for rEnal denervation (ACHIEVE) study was a prospective, multicenter, single-arm study evaluating the safety and efficacy of uRDN (*n* = 96) [10]. Throughout 12 months post-uRDN, 24 h ambulatory SBP decreased by 7.5 mmHg in the absence of any safety issues [10]. With the exception of the long-term follow-up of the pivotal uRDN studies demonstrating a sustained BP-lowering effect up to 3 years, there is a paucity of data on the long-term safety and efficacy of the therapy [11–14]. The relevance of the latter can be illustrated by questions regarding long-term deterioration of renal function, occurrence of renal artery stenosis and potential signs of renal nerve regeneration, a phenomenon previously observed in animal models [15].

The aim of the current study was to assess the long-term safety and efficacy of uRDN in patients with resistant hypertension who were initially enrolled in the ACHIEVE study.

## Methods

### Study design and population

The current prospective observational multicenter study included patients previously treated with uRDN using the Paradise system (ReCor Medical, Palo Alto, California, United States of America) within the context of the international ACHIEVE study [10]. In brief, 96 adult patients with office SBP ≥160 mmHg and 24 h ambulatory SBP ≥130 mmHg despite the use of 3 or more antihypertensive drugs, including a diuretic, were included between January 2013 and December 2014 and followed-up for 12 months. Exclusion criteria were renal artery anatomy ineligible for uRDN (main renal artery length <20 mm, main renal artery diameter <4 mm), renal artery stenosis or an *estimated Glomerular Filtration Rate* (eGFR) <45 ml/min/1.73m^2^.

Within the current long-term follow-up study, all patients provided informed consent for the use of their data and the study was conducted in accordance with the Declaration of Helsinki. Local ethical approval was obtained in all countries (Sweden, Germany, The Netherlands) and the study was registered in the German Clinical Trials Register (DRKS-ID: DRKS00029639).

### Study procedures and data collection

All eight sites involved in the initial study were approached and four sites agreed to participate in the current long-term follow-up study. Participating sites invited all patients still alive at the time of follow-up, to a prospective outpatient clinic study visit, consisting of 24 h ambulatory BP and office BP measurements, renal function testing and data collection on medication regimen and adverse events. Data collected at these visits were used for the primary efficacy analysis.

**Fig. 1.**
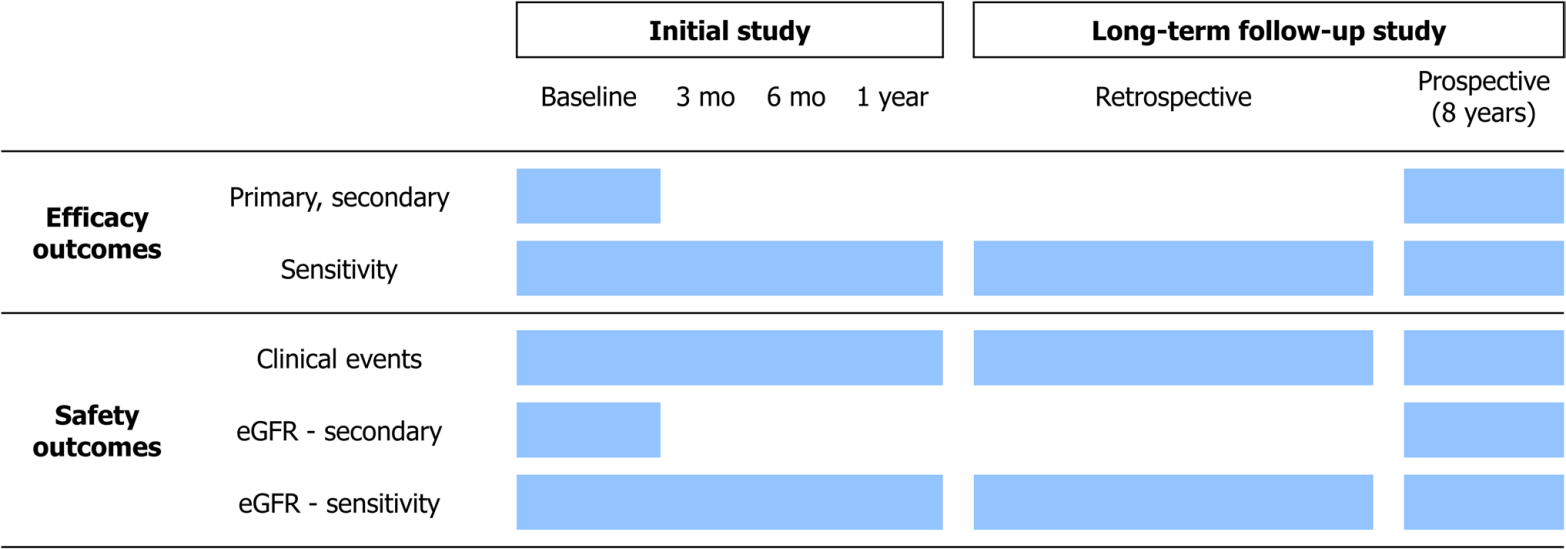
Data collection and study outcomes. *eGFR, estimated Glomerular Filtration Rate*

Retrospective follow-up data were collected in all patients following the end of follow-up of the initial study (12 months post-uRDN). These data involved ambulatory BP, office BP, renal function testing, medication regimen and adverse events. In patients who declined participation in prospective follow-up visits or those initially enrolled in sites that were not willing to participate in active long-term follow-up, efforts were made to collect safety and efficacy data up to the longest available follow-up moment. Patients who declined a prospective visit were asked to provide informed consent only for the use of their retrospective follow-up data (Fig. 1).

### Outcomes

The primary efficacy outcome was the change in 24 h ambulatory SBP between baseline (pre uRDN) and 8-year follow-up. Secondary efficacy outcomes were the changes in 24 h ambulatory diastolic blood pressure (DBP), daytime ambulatory BP, nighttime ambulatory BP, office BP, the number of defined daily dosages (DDD) and the number of classes of antihypertensive drugs between baseline (pre uRDN) and 8-year follow-up [16].

The primary safety outcome was a composite endpoint, consisting of all-cause mortality, any embolic event causing end-organ damage (including myocardial infarction and stroke), renal failure (defined as eGFR<15 ml/min per 1.73 m^2^ or need for dialysis), new renal artery stenosis of ≥70% and hospitalization for hypertensive crisis. Secondary safety outcomes were the individual components of the primary safety outcome and the change in renal function (eGFR) between baseline and 8 years.

### Sensitivity analyses

For all efficacy outcomes, the evolution over time was studied by adding follow-up data from the initial study and retrospective follow-up data to the analyses. A similar approach was used for the safety outcome renal function (Fig. 1).

### Statistical analysis

Categorical variables were expressed as counts (percentages). Continuous variables were described as mean ± standard deviation (SD), or as median [25^th^–75^th^ percentile], depending on variable distributions. Normality was assessed using quantile-quantile plots and the Shapiro–Wilk test.

The primary efficacy outcome was analyzed in patients that participated in the prospective outpatient clinic study visit, using a linear mixed-effects model to account for between-patient differences in follow-up time. The dependent variable was 24 h ambulatory SBP and fixed effects were used for follow-up time and the number of DDDs of antihypertensive drugs. Random intercepts were used to account for repeated measurements (baseline and prospective follow-up visit) within patients, while random slopes for follow-up time were included only if they significantly improved the model fit. Inverse probability weighting was applied to adjust for loss-to-follow-up and weights were calculated based on age, sex, body mass index, medical history (smoking, diabetes, dyslipidemia, cardiovascular events), renal function (eGFR), 24 h ambulatory SBP and DBP and the number of DDDs of antihypertensive drugs at baseline. To facilitate interpretation of the regression coefficient of the fixed effect of time as the modeled 8-year change in the outcome variable, follow-up time was scaled to an 8-year time horizon in the regression model. This regression coefficient, including the corresponding 95% confidence interval (CI) and P value, was reported.

The primary safety outcome (i.e., the composite outcome) and secondary event outcomes (i.e., the individual components of the composite outcome) were analyzed using Kaplan–Meier analysis. All patients from the ACHIEVE initial study (follow-up 12 months) were included in the survival analyses, irrespective of whether or not they participated in the current long-term follow-up study (prolonged follow-up up until 8 years). The total number of events and the Kaplan–Meier estimate for the cumulative incidence (including the 95% CI) were reported.

The 8-year changes in continuous secondary efficacy outcomes (i.e., 24 h ambulatory DBP, daytime/nighttime ambulatory BP, office BP, antihypertensive drug DDDs and classes) and safety outcomes (i.e., renal function) were analyzed using a similar approach as for the primary efficacy outcome. Fixed effects for the number of DDDs of antihypertensive drugs were only used in models estimating BP outcomes.

In the sensitivity analyses, the evolution of continuous efficacy outcomes (i.e., 24 h/daytime/nighttime ambulatory BP, office BP, antihypertensive drug DDDs and classes) and safety outcomes (i.e., renal function) over time was analyzed using a similar approach as for the primary efficacy outcome. However, follow-up data from the initial study (up until 12 months post-uRDN) and retrospective follow-up data (between 12 months post-uRDN and the prospective follow-up visit) were added to the model. The linear effect of follow-up time was replaced by a non-linear effect (using natural splines with 3 degrees of freedom) in case this significantly improved the model fit. Results were displayed using effect plots and P values for the overall change in the outcome variable over time.

As the maximal sample size in this long-term study was limited to the number of participants in the initial study (*n* = 96), no formal sample-size calculation was performed. In general, two-sided P values <0.05 were considered statistically significant. Analyses were performed using R version 4.3.0 with packages “ipw”, “nlme”, “splines”, “survival” and “ggplot2” [17].

## Results

### Study population

**Fig. 2.**
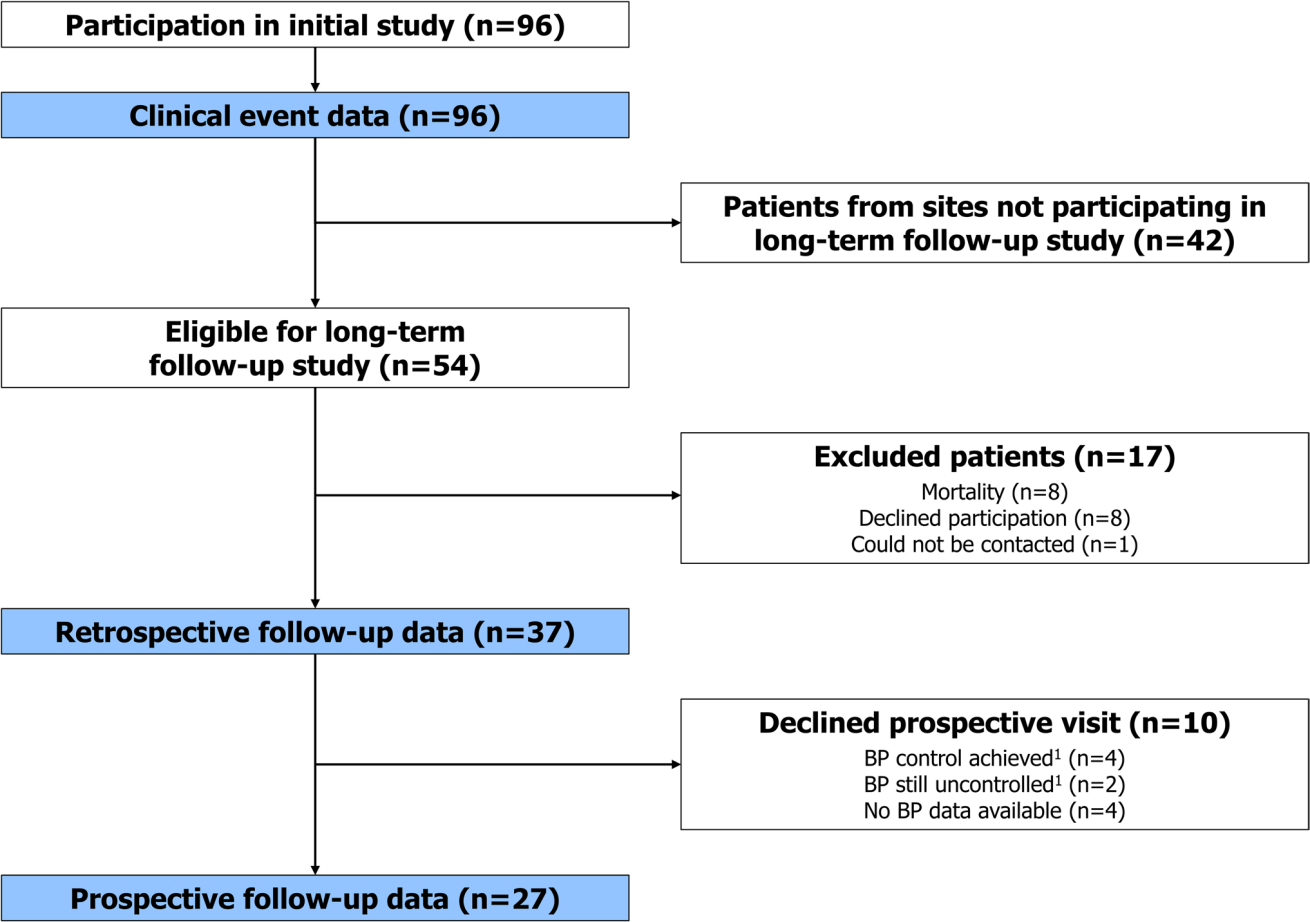
Study flowchart. ^1^Defined as assessed by investigator’s discretion

A total of 96 patients were treated with uRDN in context of the initial ACHIEVE study. Out of these, 54 patients were eligible for participation in the present long-term follow-up study, based on the willingness of sites to participate in the current study. Retrospective follow-up data (> 12 months) was available for 37 patients (69%) and 27 patients completed a prospective follow-up visit (50%) with a median follow-up duration of 8.2 [25^th^–75^th^ percentile 7.6–8.9] years (Fig. 2). Out of all eligible patients (*n* = 54), baseline characteristics were comparable between patients who completed a prospective follow-up (*n* = 27) visit vs. those who did not (*n* = 27; Supplemental Table 1). Similarly, the change in mean 24 h ambulatory SBP at 12 months (the primary outcome of the initial ACHIEVE study) did not differ between patients with and without a prospective follow-up visit (−7.9 ± 16.5 vs. −7.2 ± ± 19.3; *P* = 0.86). All patients from the initial ACHIEVE study were included in the safety and sensitivity analyses (*n* = 96).

Within patients with a prospective follow-up visit, mean age at the time of the index procedure was 62.6 ± 9.3 years and 10 (37.0%) patients were female. Preprocedural 24 h ambulatory BP was 151.9/84.1 ± 11.5/11.1 mmHg and office BP was 178.1/93.1 ± 18.0/13.3 mmHg, while patients were prescribed a median of 5.0 [25^th^–75^th^ percentile 4.3–7.0] DDDs of antihypertensive drugs. During the index procedure, patients received a median number of 6 [25^th^–75^th^ percentile 4–6] renal artery ablations bilaterally (Table 1).

**Table 1:** Baseline characteristics

	Prospective follow-up data available (*n* = 27)	Clinical event data available (*n* = 96)
Clinical parameters		
Age (years), mean ± SD	62.6 ± 9.3	64.0 ± 10.3
Female sex, n (%)	10 (37.0)	39 (40.6)
Body mass index (kg/m^2^), median [25^th^-75^th^ percentile]	29.4 [26.8−34.0]	29.0 [25.9−33.1]
Cardiovascular risk factors		
Current smoker, n (%)	6 (22.2)	9 (9.4)
Ever-smoker, n (%)	9 (33.3)	39 (40.6)
Diabetes, n (%)	9 (33.3)	38 (39.6)
Dyslipidemia, n (%)	16 (59.3)	51 (53.1)
Medical history		
Myocardial infarction, n (%)	9 (33.3)	23 (24.0)
Coronary revascularization, n (%)	7 (25.9)	25 (26.0)
Stroke, n (%)	1 (3.7)	10 (10.4)
Heart failure, n (%)	0 (0.0)	6 (6.3)
Peripheral vascular disease, n (%)	1 (3.7)	8 (8.3)
Obstructive sleep apnea, n (%)	4 (14.8)	20 (20.8)
Renal function		
Estimated glomerular filtration rate (ml/min/1.73m^2^), mean ± SD	81.0 ± 17.3	76.7 ± 18.8
Blood pressure		
24 h ambulatory blood pressure (mmHg), mean ± SD	151.9/84.1 ± 11.5/11.1	156.2/88.4 ± 15.4/12.7
Daytime ambulatory blood pressure (mmHg), mean ± SD	155.4/87.9 ± 11.4/11.7	158.9/91.6 ± 16.1/13.4
Nighttime ambulatory blood pressure (mmHg), mean ± SD	142.7/76.3 ± 16.5/10.8	146.9/80.4 ± 17.7/13.0
Office blood pressure (mmHg), mean ± SD	178.1/93.1 ± 18.0/13.3	176.5/95.2 ± 20.7/15.7
Antihypertensive medication		
Number of defined daily dosages, median [25^th^–75^th^ percentile]	5.0 [4.3−7.0]	5.4 [4.1−7.4]
Number of classes, median [25^th^–75^th^ percentile]	4 [3–4]	4 [3–5]
Procedural characteristics		
Procedure duration (minutes), median [25^th^–75^th^ percentile]	56.5 [40.0−72.8]	50.0 [38.0−66.5]
Total renal artery ablations, median [25^th^–75^th^ percentile]	6 [4–6]	5 [4–6]
Left renal artery ablations, median [25^th^–75^th^ percentile]	3 [2–3]	2 [2–3]
Right renal artery ablations, median [25^th^–75^th^ percentile]	3 [2–3]	2 [2–3]

#### Efficacy outcomes

**Fig. 3.**
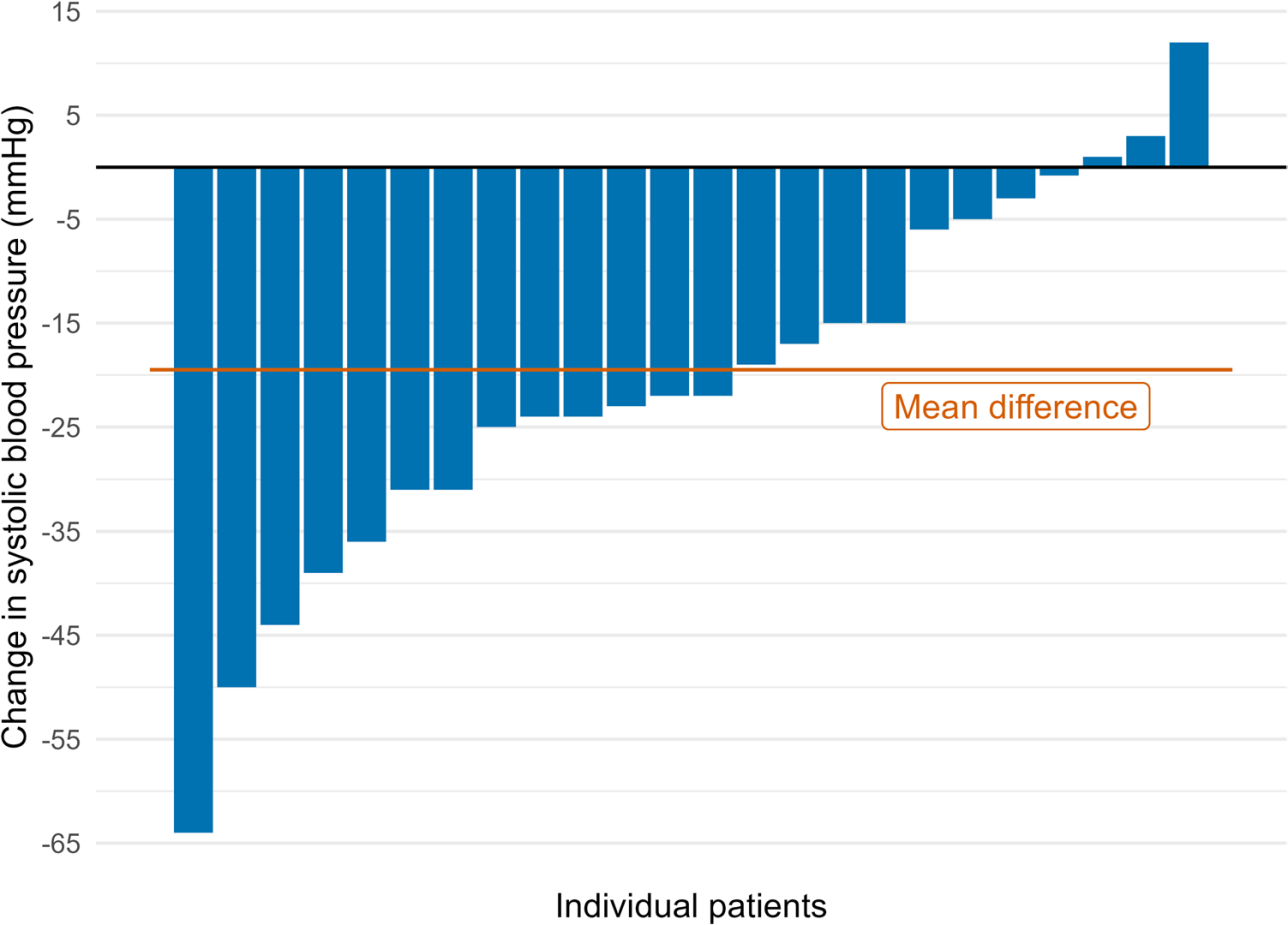
Change in mean 24 h ambulatory systolic blood pressure in individual patients between baseline and 8-year follow-up

At 8-year follow-up, the modeled change in 24 h ambulatory SBP was −19.5 [95% CI −26.7, −12.4] mmHg (*P*<0.001) following adjustment for the number of antihypertensive drug DDDs over time (Fig. 3). Similarly, 24 h ambulatory DBP changed −9.5 [95% CI −14.5, −4.4] mmHg (*P* = 0.001). Office SBP changed −22.1 [95% CI −34.3, −9.8] mmHg (*p* = 0.001) and office DBP changed −11.2 [95% CI −16.9, −5.5] mmHg (*P* = 0.001). Similar reductions were observed for daytime and nighttime ambulatory BP. The number of DDDs of antihypertensive drugs reduced by −1.7 [95% CI −2.8, −0.6] as compared to baseline (*P* = 0.003) (Table 2). Unadjusted outcome measures have been displayed in Supplemental Table 2.

**Table 2:** Estimated 8-year changes in blood pressure, antihypertensive medication and renal function in patients who completed a prospective follow-up visit (*n* = 27)

	Effect estimate^1^	95% confidence interval	*P* value
Blood pressure^2^			
24 h ambulatory systolic blood pressure (mmHg)	−19.5	−26.7, −12.4	<0.001
24 h ambulatory diastolic blood pressure (mmHg)	−9.5	−14.5, −4.4	0.001
Daytime ambulatory systolic blood pressure (mmHg)	−21.6	−29.0, −14.3	<0.001
Daytime ambulatory diastolic blood pressure (mmHg)	−12.1	−17.7, −6.6	<0.001
Nighttime ambulatory systolic blood pressure (mmHg)	−13.4	−22.1, −4.7	0.004
Nighttime ambulatory diastolic blood pressure (mmHg)	−5.4	−10.3, −0.5	0.03
Office systolic blood pressure (mmHg)	−22.1	−34.3, −9.8	0.001
Office diastolic blood pressure (mmHg)	−11.2	−16.9, −5.5	0.001
Antihypertensive medication			
Number of defined daily dosages	−1.7	−2.8, −0.6	0.003
Number of classes	−0.3	−0.9, 0.2	0.21
Renal function			
Estimated glomerular filtration rate (ml/min/1.73m^2^)	−8.9	−13.2, -4.7	<0.001

^1^Inverse probability weighting has been implemented in all analyses to adjust for loss-to-follow-up

^2^All blood-pressure outcomes analyses have been adjusted for changes in defined daily dosages of antihypertensive drugs over time

**Fig. 4.**
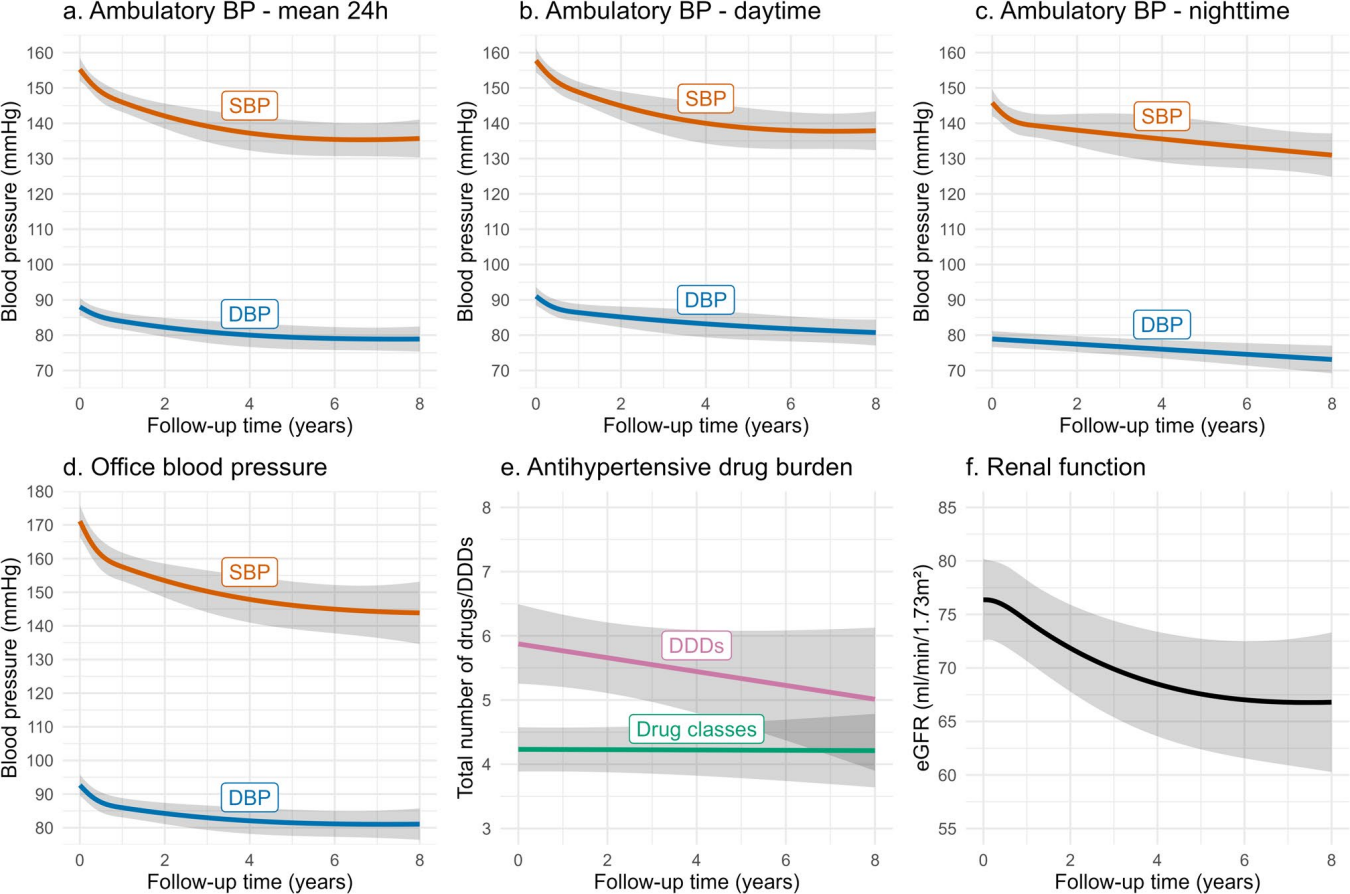
Sensitivity analyses on evolution of blood pressure, antihypertensive drug burden and renal function following ultrasound renal denervation. *BP*, blood pressure. *DBP*, diastolic blood pressure. *DDD*, defined daily dosage. *eGFR*, estimated Glomerular Filtration Rate. *SBP*, systolic blood pressure

The findings above were confirmed in the sensitivity analyses, incorporating also follow-up data from the initial study and retrospective follow-up data (96 patients, 608 observations). Significant reductions were observed in 24 h ambulatory SBP (*P*<0.001) and DBP (*P*<0.001), daytime ambulatory SBP (*p*<0.001) and DBP (*P*<0.001), nighttime ambulatory SBP (*P*<0.001) and DBP (*P* = 0.002) and office SBP (*P*<0.001) and DBP (*P*<0.001) (Fig. 4a–d). In contrast to patients who completed a prospective follow-up visit, the number of antihypertensive drug DDDs (*P* = 0.18) and drug classes (*P* = 0.94) remained stable over time (Fig. 4e).

#### Safety outcomes

Clinical event data were available for 96 patients with median follow-up time of 3.5 [25^th^–75^th^ percentile 1.0–8.0] years. The primary safety outcome evaluated the composite rate of prespecified clinical events of interest. Clinical events of interest occurred in 27 patients (cumulative incidence 41.0% [95% CI 26.0%–52.9%]). The most common individual components of the composite outcome were all-cause mortality (8 cases; cumulative incidence 17.1% [95% CI 4.7%–27.9%] at 10 years) and hypertensive crisis (14 cases; cumulative incidence 21.4% [95% CI 8.7%–32.3%] at 10 years) (Table 3).

**Table 3:** Clinical event outcomes

	Number of events (*n* = 96)	Cumulative incidence at 10 years (%)^1^	Cumulative incidence - 95% confidence interval (%)^1^
Composite outcome	27	41.0	26.0 – 52.9
Individual components of composite outcome			
All-cause mortality	8	17.1	4.7 – 27.9
Stroke	3	7.5	0.0 – 15.8
Myocardial infarction	4	7.1	0.0 – 14.8
Other thromboembolic event	0	0.0	0.0 – 0.0
Renal failure^2^	1	3.6	0.0 – 10.2
Renal artery stenosis	0	0.0	0.0 – 0.0
Hypertensive crisis	14	21.4	8.7 – 32.3

^1^Cumulative incidence estimates (including confidence intervals) were derived from the Kaplan–Meier curve

^2^Defined as an estimated Glomerular Filtration Rate ≤ 15 ml/min/1.73m^2^ and/or requirement of dialysis

Within patients who completed a prospective follow-up visit, the 8-year modeled decline in eGFR was −8.9 [95% CI −13.2, −4.7] ml/min/1.73m^2^ (*P*<0.001) (Table 2). These findings were consistent when compared to a sensitivity analysis which also included initial study follow-up data and retrospective follow-up data (*P*<0.001; Fig. 4f).

Renal failure occurred in one patient (cumulative incidence 3.6% [95% CI 0.0%–10.2%] at 10 years). This individual patient had a baseline eGFR of 50 ml/min/1.73m^2^ which initially improved to 64 ml/min/1.73m^2^ throughout 2 years following the procedure. Afterward, renal function declined and dialysis was required at 5 years post-uRDN. No events of renal artery stenosis were reported (Table 3).

## Discussion

The present study is among the first to address long-term clinical event and BP outcomes in patients previously treated with uRDN. We were able to demonstrate a sustained reduction in each of the individual BP components accompanied by a decline in antihypertensive drug burden. No procedure-related serious adverse events occurred throughout the course of the study.

In patients at a median of 8 years after uRDN, we observed a sustained reduction in 24 h ambulatory SBP of 19.5 mmHg after adjustment for antihypertensive drug burden. In parallel, the number of DDDs of antihypertensive drugs decreased by 1.7 during follow-up. This finding is most likely to be explained by dose reductions rather than discontinuation of particular antihypertensive drug classes, as the number of drug classes remained stable over time. In contrast to previous sham-controlled randomized studies on uRDN, no standardized medical therapy was embedded within the initial ACHIEVE study protocol and long-term follow-up studies [6–8, 10]. Patients therefore received medical therapy as per local physician’s discretion. As a result, the present study provides novel insights on the reduction in BP in real-world patients with resistant hypertension who underwent uRDN 8 years ago.

The positive results of multiple sham-controlled RDN trials have increased the interest in long-term studies evaluating the safety and efficacy of the therapy [6–9, 18, 19]. However, there is a paucity of long-term follow-up data, and available studies have been complicated by loss-to-follow-up [20]. The present study aimed to overcome these limitations by re-inviting all patients treated with uRDN within the context of the initial ACHIEVE study to collect 8-year safety and efficacy data. Within our study, inverse probability weighting was applied to adjust for any effect of loss-to-follow-up on the study’s outcomes. Furthermore, sensitivity analyses were performed, in which data from the prospective follow-up visits were pooled with data from the initial ACHIEVE study and retrospectively collected follow-up data (including a total of 608 BP observations). For both ambulatory and office BP outcomes, these analyses demonstrated that the most pronounced reductions occurred within the first year following uRDN. Between one and five years, smaller additional BP reductions were observed. These findings are in line with other previous long-term follow-up data [20, 21]. The latter supports the rationale for long-term follow-up studies (taking into account changes in antihypertensive drug load and compliance over time). Overall, the sensitivity analyses confirmed the robustness of our findings on BP reduction and eGFR change throughout follow-up.

With respect to the number of antihypertensive drug DDDs, conflicting results were observed as drug burden declined in patients who completed a prospective follow-up visit, whereas it remained stable over time in the sensitivity analyses. This finding is likely to be explained by a confounding-by-indication phenomenon, as the retrospective follow-up data were derived from visits that were scheduled based on a clinical indication. In turn, this could have resulted in a relationship between the availability of the data and the outcomes of this study. More specifically, patients with a higher need for uptitration of their antihypertensive medication are likely to have had more visits to their hypertension specialist, thereby contributing relatively more to the data collection in the sensitivity analyses of this study. That said, the primary efficacy outcome analyses (58 observations) as well as the sensitivity analyses (608 observations) confirmed a durable BP reduction over time following uRDN, without any signs of an increase in antihypertensive drug burden.

As mentioned above, limited data is available on the long-term follow-up after uRDN. One recent study demonstrated an 8.4 mmHg reduction in office SBP (as compared to preprocedural screening) at 3 years while patients were on a similar number of antihypertensive drugs [14]. A combined registry of radiofrequency-based RDN (rfRDN) and uRDN patients demonstrated a reduction in daytime ambulatory SBP of 20.9 mmHg at 5 years, while antihypertensive drug burden remained stable over time [21]. Specifically related to rfRDN, reductions in mean 24 h ambulatory SBP of 12.1 and 16.2 mmHg were observed at 9 and 10 years follow-up, while medication burden decreased or remained stable, respectively [22, 23]. Within the present study, a reduction in 24 h ambulatory SBP of 19.5 mmHg was observed at 8 years following uRDN, in parallel to a 1.7 decline in the number of antihypertensive drug DDDs. Direct comparisons of our findings to previous literature are hampered by differences in study design, including discrepancies regarding study population, RDN treatment modality and follow-up duration. That said, the present study supports previous publications reporting sustained BP reductions in the absence of intensified antihypertensive drug treatment over time following RDN.

Within the current study, no serious uRDN procedure-related adverse events occurred. The cumulative incidence of the composite outcome reached 41.0% at 10 years, which could be explained by substantial rates of hypertensive crisis (21.4%), stroke (7.5%) and mortality (17.1%). These findings are indicative of the high cardiovascular risk of our study population, which consisted of patients with resistant hypertension and a substantial number of baseline risk factors and previous cardiovascular events. Of interest, and although no protocol-mandated noninvasive renal artery imaging was performed at long-term follow-up, no cases of renal artery stenosis were observed. Therefore, underreporting of subclinical cases of renal artery stenosis could not be ruled out. Nonetheless, only one patient with pre-existent chronic kidney disease suffered from incident renal failure and required dialysis 5 years post-RDN. Overall, renal function (eGFR) declined by 8.9 ml/min/1.73m^2^ within an 8-year time window, equal to a decline of 1.1 ml/min/1.73m^2^ per year. These findings were confirmed in the sensitivity analysis also taking into account retrospectively collected data. Overall, the magnitude of the decline in renal function (eGFR) was in line with previous studies, which reported effect sizes ranging from 0.9 to 2.1 ml/min/1.73m^2^ per year [20–22, 24]. These findings correspond to the natural course of renal function in hypertensive patients and thereby indicate preservation of renal function on the long term in patients with resistant hypertension who are treated with uRDN [25].

### Limitations

This study has several limitations. First, the current study lacked a (sham) control arm, which precludes any statements on causality between the uRDN procedure and the study outcomes. Second, a total of 42 patients from the initial ACHIEVE study were not included in this long-term follow-up study as their treating sites declined participation for administrative or logistical reasons (e.g., no research facilities available, discontinuation of the RDN research program). Patients treated at these centers were considered missing completely at random as all reasons were considered unrelated to outcomes in individual patients [26]. Out of the remaining 54 patients, retrospective and prospective follow-up data was collected in 37 (69%) and 27 patients (50%), respectively. Adjustment for loss-to-follow-up was performed using inverse probability weighting, based on a comprehensive set of baseline characteristics that are known to affect future cardiovascular risk. However, we cannot rule out selective loss-to-follow-up based on unmeasured confounding variables, as well as a limited level of precision due to the decreased sample size (as compared to the ACHIEVE initial study). To overcome these limitations, future RDN studies should incorporate protocolized long-term follow-up. Third, while clinical event data was analyzed for all patients from the ACHIEVE initial study (*n* = 96), the median follow-up duration for clinical events was only 3.5 years, which resulted in a limited level of precision of the cumulative incidence estimates. Lastly, this study lacked therapy adherence testing. Therefore, we cannot rule out that changes in therapy adherence throughout the study have influenced our results.

## Conclusions

Throughout 8 years following uRDN, a significant reduction in ambulatory BP and antihypertensive drug burden was observed in patients with resistant hypertension. Within this study, no procedure-related adverse events occurred. These findings support the use of uRDN as an adjunctive therapy for the treatment of hypertension. Future (randomized) studies including protocolized long-term follow-up visits will provide more insights on the safety and the efficacy of uRDN on the long term.

## Supplementary Information

Below is the link to the electronic supplementary material.

Supplementary file1 (PDF 101 kb)
