# Supplementary material for: Long-term safety and efficacy of endovascular ultrasound renal denervation in resistant hypertension: 8-year results from the ACHIEVE study
Source: Clin Res Cardiol. 2024 Oct 23;115(8):1327–36. doi: 10.1007/s00392-024-02555-7 (PMC13346321; doi:10.1007/s00392-024-02555-7)
Supplement: Supplementary file 1 — Supplementary file1 (PDF 101 kb) [file 392_2024_2555_MOESM1_ESM.pdf]

Supplemental Material

Supplemental Table 1. Sensitivity analyses comparing baseline characteristics between patients with and without a prospective long-term follow-up visit for centers who participated in the long-term follow-up study

	**Patients with prospective long-term follow-up (n=27)**	**Patients without prospective long-term follow-up (n=27)**	**P-value**
**Clinical parameters**			
Age (years), mean ± SD	62.6 ± 9.3	64.6 ± 10.6	0.47
Female sex, n (%)	10 (37.0)	13 (48.1)	0.59
Body mass index (kg/m^2), median [25th-75th percentile]	30.7 ± 5.9	29.4 ± 5.9	0.41
**Cardiovascular risk factors**			
Current smoker, n (%)	6 (22.2)	2 (7.4)	<0.001
Ever-smoker, n (%)	9 (33.3)	18 (66.7)	
Diabetes, n (%)	9 (33.3)	14 (51.9)	0.27
Dyslipidemia, n (%)	16 (59.3)	14 (51.9)	0.78
**Medical history**			
Myocardial infarction, n (%)	9 (33.3)	4 (14.8)	0.20
Coronary revascularization, n (%)	7 (25.9)	6 (22.2)	1.00
Stroke, n (%)	1 (3.7)	3 (11.1)	0.61
Heart failure, n (%)	0 (0.0)	3 (11.1)	0.24
Peripheral vascular disease, n (%)	1 (3.7)	0 (0.0)	1.00
Obstructive sleep apnea, n (%)	4 (14.8)	5 (18.5)	1.00
**Renal function**			
Estimated glomerular filtration rate (ml/min/1.73m^2), mean ± SD	81.0 ± 17.3	75.8 ± 19.8	0.31

**Blood pressure**			
24h ambulatory systolic blood pressure (mmHg), mean ± SD	151.9 ± 11.5	155.7 ± 16.1	0.32
24h ambulatory diastolic blood pressure (mmHg), mean ± SD	84.1 ± 11.1	86.8 ± 14.1	0.44
Daytime ambulatory systolic blood pressure (mmHg), mean ± SD	155.4 ± 11.4	158.9 ± 16.4	0.37
Daytime ambulatory diastolic blood pressure (mmHg), mean ± SD	87.9 ± 11.7	89.7 ± 14.6	0.60
Nighttime ambulatory systolic blood pressure (mmHg), mean ± SD	142.7 ± 16.5	146.9 ± 19.1	0.40
Nighttime ambulatory diastolic blood pressure (mmHg), mean ± SD	76.3 ± 10.8	79.3 ± 14.6	0.39
Office systolic blood pressure (mmHg), mean ± SD	178.1 ± 18.0	173.5 ± 19.7	0.38
Office diastolic blood pressure (mmHg), mean ± SD	93.1 ± 13.3	93.6 ± 18.1	0.89
**Antihypertensive medication**			
Number of defined daily dosages, median [25th-75th percentile]	5.0 [4.3-7.0]	5.6 [4.6-7.2]	0.63
Number of classes, median [25th-75th percentile]	4 [3-4]	5 [3-5]	0.25
**Procedural characteristics**			
Procedure duration (minutes), median [25th-75th percentile]	56.5 [40.0-72.8]	42.0 [39.0-60.0]	0.36
Total renal artery ablations, median [25th-75th percentile]	6 [4-6]	5 [4-6]	0.052
Left renal artery ablations, median [25th-75th percentile]	3 [2-3]	2 [2-3]	0.07

Right renal artery ablations, median [25th-75th percentile]	3 [2-3]	2 [2-3]	0.09

Supplemental Table 2. Estimated *unadjusted* 8-year changes in blood pressure, antihypertensive medication and renal function in patients who completed a prospective follow-up visit (n=27).

	**Mean difference**	**95% confidence interval**	**P-value[1]**
**Blood pressure[2]**			
24h ambulatory systolic blood pressure (mmHg)	-20.8	-28.4, -13.2	<0.001
24h ambulatory diastolic blood pressure (mmHg)	-8.9	-13.8, -4.1	0.001
Daytime ambulatory systolic blood pressure (mmHg)	-22.7	-30.9, -14.4	<0.001
Daytime ambulatory diastolic blood pressure (mmHg)	-11.5	-16.8, -6.2	<0.001
Nighttime ambulatory systolic blood pressure (mmHg)	-16.1	-24.5, -7.7	0.001
Nighttime ambulatory diastolic blood pressure (mmHg)	-4.8	-9.7, 0.0	0.052
Office systolic blood pressure (mmHg)	-35.6	-48.7, -22.5	<0.001
Office diastolic blood pressure (mmHg)	-12.9	-20.2, -5.7	0.001
**Antihypertensive medication**			
Number of defined daily dosages	-1.9	-3.7, -0.2	0.01
Number of classes	-0.3	-0.9, 0.3	0.41
**Renal function**			
Estimated glomerular filtration rate (ml/min/1.73m^2)	-11.8	-17.3, -6.3	<0.001

[1] Statistical testing was performed using the paired t-test or Wilcoxon signed rank test for differences with a normal or a skewed distribution, respectively.
